# A THEORETICAL EXAMINATION INTO THE CONNECTIONS BETWEEN LINKED LIVES AND LATER LIFE DISABILITIES

**DOI:** 10.1093/geroni/igac059.2718

**Published:** 2022-12-20

**Authors:** Akwasi Adjei Gyimah, Samuel Van Vleet

**Affiliations:** Miami University, Oxford, Ohio, United States; Miami University, Oxford, Ohio, United States

## Abstract

As people age, they find themselves living similar lives to those around them. This concept is often referred to as linked lives. Linked lives explains that individual lives are often interdependent. This is due to various elements such as social and historical influences. These influences can imprint networks of shared relationships. Researchers have found that the first point of socialization as humans are social relationships with family and friends. However, while the connections of linked lives are often attributed to early life course trajectories, little research has evaluated the interdependency that is created with disabilities in later life. Life course disability research is a key factor of gerontology because a majority of older adults encounter disabilities within later life. This attributed disability can often forge bound and interdependence within people and their own social networks. This paper theoretically examines different perspectives of disabilities research and how it relates to the fundamental principles of linked lives. This paper highlights the gap in the current gerontological understanding of the later life links among older adults with disabilities. This theory paper provides the needed information for gerontologist focusing on older people with disabilities to be able to evaluate allocations needed for providing adequate social support and services tailored towards the needs of these interdependent lives.

